# Meningoencephalitis, coronary artery and keratitis as an onset of brucellosis: a case report

**DOI:** 10.1186/s12879-020-05358-z

**Published:** 2020-09-07

**Authors:** Lingling Geng, Yuan Feng, Dan Li, Nan Nan, Kai Ma, Xianyan Tang, Xiaoqing Li

**Affiliations:** Department of Rheumatology and Immunology, Xi’an Children’s Hospital Affiliated to Xi’an Jiaotong University, Xi’an, 710003 People’s Republic of China

**Keywords:** Brucella, Brucellosis, Meningoencephalitis, Coronary artery, mNGS, Case report

## Abstract

**Background:**

Brucellosis is a zoonotic disease caused by brucella. It has been an increasing trend in recent years (Wang H, Xu WM, Zhu KJ, Zhu SJ, Zhang HF, Wang J, Yang Y, Shao FY, Jiang NM, Tao ZY, Jin HY, Tang Y, Huo LL, Dong F, Li ZJ, Ding H, Liu ZG, Emerg Microbes Infect 9:889-99, 2020). Brucellosis is capable to invade multiple systems throughout the body, lacking in typical clinical manifestations, and easily misdiagnosed and mistreated.

**Case presentation:**

We report a case of a male, 5-year-and-11-month old child without relevant medical history, who was admitted to hospital for 20 days of fever. When admitted to the hospital, we found that he was enervated, irritable and sleepy, accompanied with red eyes phenomenon. After anti-infection treatment with meropenem, no improvement observed. Lumbar puncture revealed normal CSF protein, normal cells, and negative culture. Later, doppler echocardiography suggested coronary aneurysms, and incomplete Kawasaki Disease with coronary aneurysms was proposed. The next day, brucellosis agglutination test was positive. Metagenomic next-generation sequencing (mNGS) of cerebrospinal fluid suggested B.melitensis, which was confirmed again by blood culture. The child was finally diagnosed as brucellosis with meningocephalitis, coronary aneurysm and keratitis. According to our preliminary research and review, such case has never been reported in detail before. After diagnosis confirmation, the child was treated with rifampicin, compound sulfamethoxazole, and ceftriaxone for cocktail anti-infection therapy. Aspirin and dipyridamole were also applied for anticoagulant therapy. After medical treatment, body temperature of the child has reached normal level, eye symptoms alleviated, and mental condition gradually turned normal. Re-examination of the doppler echocardiographic indicated that the coronary aneurysm was aggravated, so warfarin was added for amplification of anticoagulation treatment. At present, 3 months of follow-up, the coronary artery dilatation gradually assuaged, and the condition is continued to alleviate.

**Conclusion:**

Brucellosis can invade nervous system, coronary artery, and cornea. Brucellosis lacks specific signs for clinical diagnosis. The traditional agglutination test and the new mNGS are convenient and effective, which can provide the reference for clinical diagnosis.

## Background

Brucellosis is a zoonotic disease, which causes serious health risks and economic burdens to many countries and regions in the world [1]. Studies have shown that approximately 500,000 people are suffering from brucellosis each year in the world, with the majority of cases emerging from pastoral areas and rural areas [2], and it has been an increasing trend in recent years [3]. Most people infected with brucellosis have an apparent history of exposure, such as exposure to infected animals, ingestion of brucella contaminated animal products, and inhalation of brucella containing aerosols. Brucellosis is able to cause various symptoms involving multiple systems [4] and easy to be misdiagnosed due to lack of specificity. Here, we report a male 5-year-and-11-month old Chinese child, who was diagnosed brucellosis with meningoencephalitis, coronary aneurysm and keratitis, which has not been reported before.

## Case presentation

A 5-year-and-11-month old, previously healthy Chinese child, was developed fever without obvious inducement 20 days ago. The maximum body temperature recorded was 39.4 °C, and sweating was intensified than normal condition. The child was enervated, and developed drowsiness 1 week ago, red eye phenomenon appeared 2 days ago. Physical examination showed nervous system involvement, and drowsiness and irritability occurring alternately, eye conjunctival congestion accompanied with photophobia. Nervous system examination showed hyperreflexia of the neck and positive babinski sign on the left. CT showed a slight widening of the sulcus of the brain. Upon admission, the child was preliminarily diagnosed central nervous system infection. Due to the possibility of central nervous system infection when admitted to hospital, meropenem was applied for anti-infection treatment and mannitol for reducing intracranial pressure. And the lumbar puncture examination after 2 days onwards resulted in normal physical characteristics (colorless and transparent) and normal biochemistry. Total number of white blood cells, the glucose concentration, chloride concentration and protein concentration of cerebrospinal fluids were 2.00 × 10^6^/L (normal range 0 ~ 10 × 10^6^ /L), 2.99 mmol/L(normal range 2.8 ~ 4.5 mmol/L), 125.7 mmol/L(normal range117 ~ 127 mmol /L), and 213.4 mg/L (normal range 200 ~ 400 mg /L) , respectively. CSF-Gram staining, ink staining, acid-fast staining and culture results were all negative. Other laboratory tests are shown in Table 1.

**Table 1:** Laboratory data and infection work-up

Items	Result	reference value
Sedimentation (mm/h)	72	0–20
WBC (× 10^9^/L)	3.32	4–12
Neutrophils (× 10^9^/L)	1.21	1.8–6.3
PLT (× 10^9^/L)	228	125–350
Hb (g/L)	92	115–150
Albumin (g/L)	30.9	37–51
ALT (U/L)	15	5–30
AST (U/L)	27	10–45
C-reactive protein (mg/L)	4.90	2–4
Ferritin (ng/ml)	117.80	21.81–274.66
T-SPOT	Negative	Negative
NK cell count (cells/ul)	56	90–590
TT3 (ng/ml)	1.06	1.13–1.89
IgE (IU/ml)	74.00	<60
IgM of Mycoplasma pneumoniae, legionella, adenovirus, respiratory syncytial virus, influenza a virus, influenza b virus, parainfluenza virus, rickettsial fever Q, chlamydia pneumoniae	Negative	Negative
Hepatitis virus series	Negative	Negative
HIV, EB-virus and Syphilis	Negative	Negative
Autoantibody	Negative	Negative

*PLT* Platelet, *Hb* Hemoglobin, *HIV* Human immunodeficiency virus, *ALT* Alanine aminotransferase, *AST* Aspartate aminotransferase

After 2 days onwards, doppler echocardiographic suggested left anterior descending coronary artery tumor, accompanied with inner diameter dilation of double coronary artery and apparent roughness of endometrium. Taking repeated fever, conjunctival congestion in both eyes, and poor metal condition into consideration, the possibility of incomplete Kawasaki disease with coronary aneurysm and aseptic meningoencephalitis was significant for diagnosis. Intravenous immunoglobulin was given for shock treatment with dosage 2 g/kg, while aspirin and dipyridamole were added for anti-inflammation and anti-platelet aggregation.

**Fig. 1:**
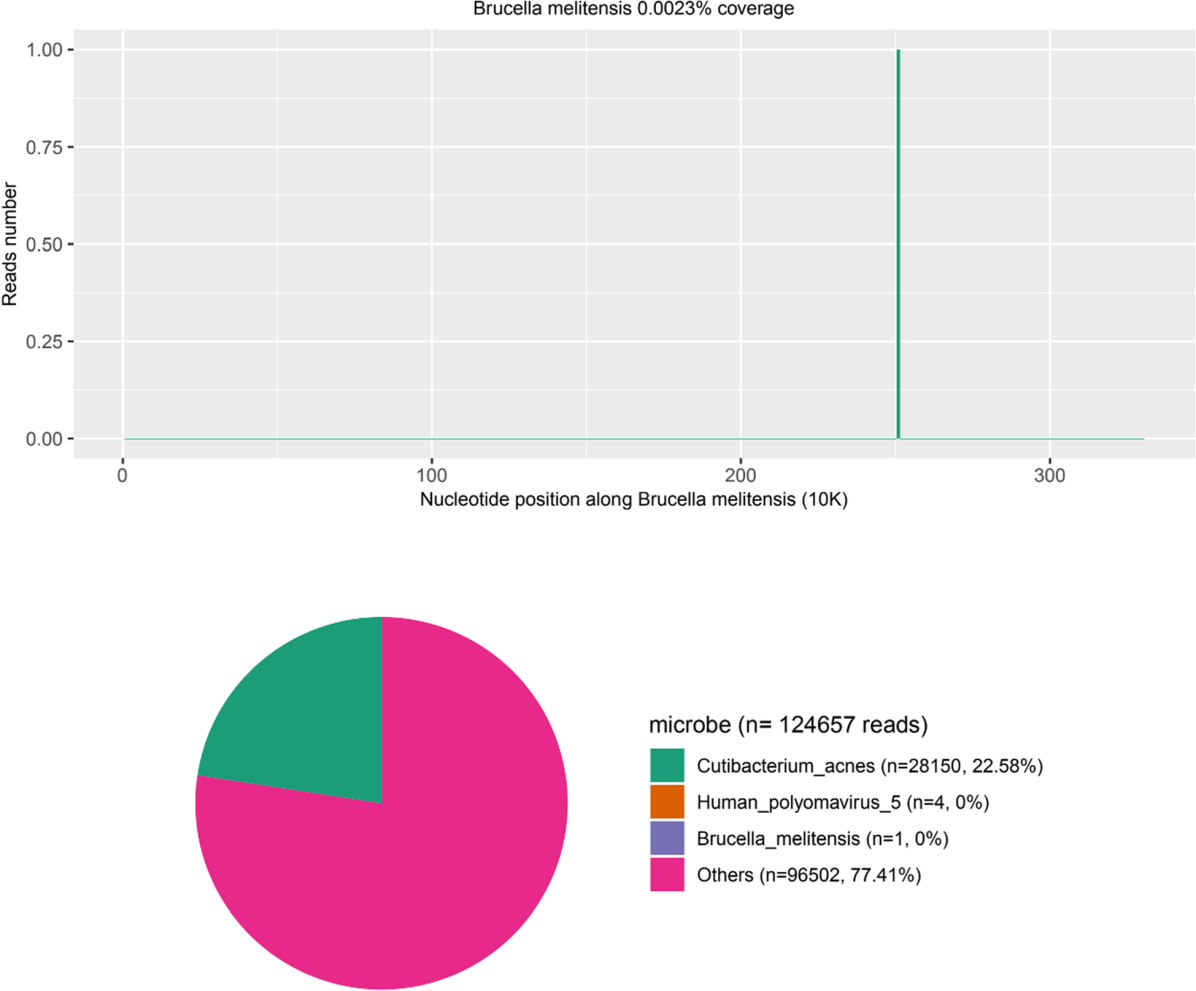
The mNGS of cerebrospinal fluid indicated br.melitensis

After 3 days onwards, brucella rose bengal precipitation test result was positive, and brucella tube agglutination test results were 1:25 (++++), 1:50 (++++), 1: 100 (++++), 1: 200 (++++), 1: 400 (+++) respectively. The result of mNGS of cerebrospinal fluid indicated br.melitensis on the same day (according to Fig. 1). Having asked again about previous medical history of the child, information of goat milk ingestion (drinking) was then learned. Through contacting the sheep owner, we have confirmed that the source individual of goat milk ingested by the child was a sick sheep. Therefore, we revised the diagnosis of brucellosis with meningoencephalitis and coronary aneurysms. After 4 days onwards, we received a report of blood culture form laboratory, in which B.melitensis was prompted. After the diagnosis of brucellosis was confirmed, the adjustment therapy was rifampicin, compound sulfamethoxazole and ceftriaxone triple anti-infection standard treatment, and aspirin and dipyridamole anticoagulant treatment. And aspirin and dipyridamole anticoagulant treatment. After treatment, body temperature of the child gradually returned to normal level and the neurological symptoms disappeared.

Another damaged organ of the child was the eyes. The conjunctiva of both eyes was obviously hyperemic and accompanied by photophobia. Ophthalmological examination revealed mild swelling of both eyelids, conjunctival congestion and edema in both eyes, altogether with corneal edema in both eyes, scattered infiltration of peripheral cornea, and positive corneal fluorescence staining. Fundus examination revealed reddish and round-shaped papillae of both eyes with unclear borders, ambiguous peripheral omentum, with no bleeding observed on the retina. IOP was normal. The ultrasonography of binocular and accessory apparatus indicated that the optic nerves of both eyes were slightly thickened. The CT of both eyes showed no obvious abnormality. With above information, diagnosis was made: 1. Keratitis in both eyes; 2. Papillary edema in both eyes.

**Fig. 2:**
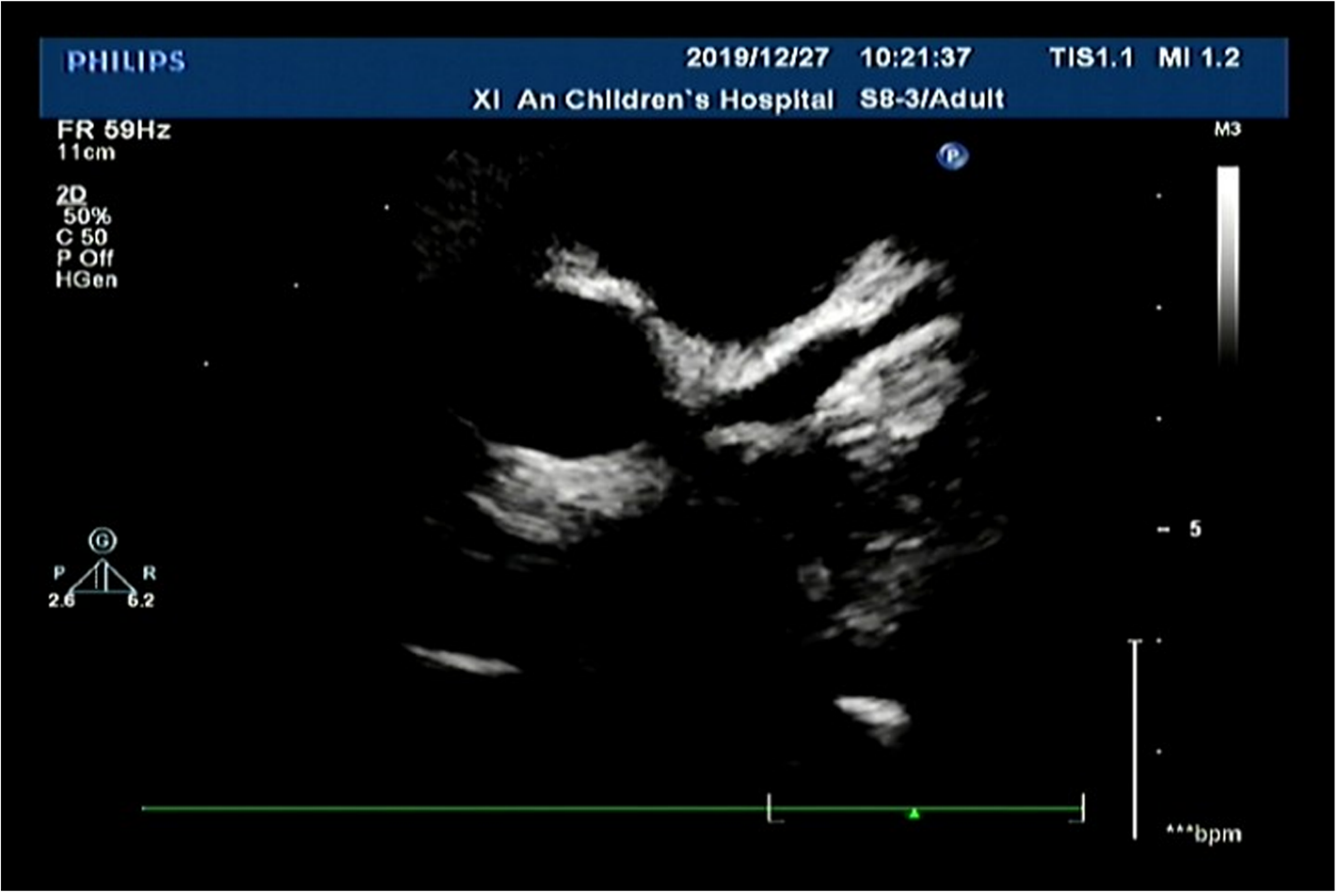
The doppler echocardiographic after 60 days onwards

After discharge, the child continued to be treated with rifampicin and compound sulfamethoxazole against brucellosis for 2 months, and continued to be treated with warfarin, aspirin and dipyridamole. Review of the doppler echocardiographic after 21 days onwards revealed the presence of multiple coronary aneurysms, warfarin anticoagulation was added. Re-examination of the doppler echocardiographic showed apparent mitigation of the child’s condition (according to Fig. 2). The result of coronary arteries with doppler echocardiographic are shown in Table 2.

**Table 2:** Inner diameter of coronary arteries by color doppler ultrasound

Days	LCA (mm)/AO (mm)	LAD (mm)/AO (mm)	RCA (mm)/AO (mm)
2	3.3/14 = 0.23	4.3/14 = 0.30	4.0/14 = 0.28
12	3.3/14 = 0.23	4.0/14 = 0.28	3.6/14 = 0.25
21	4.3/14 = 0.30	4.4/14 = 0.31	3.5/14 = 0.25
27	4.3/14 = 0.30	4.5/14 = 0.32	3.5/14 = 0.25
40	3.6/14 = 0.25	4.2/14 = 0.30	3.3/14 = 0.23
60	2.5/14 = 0.18	4.1/14 = 0.29	3.1/14 = 0.22

*LCA* Left coronary arter, *RCA* Right coronary arter, *AO* Aortic root, *LAD* Left anterior descending coronary artery

## Discussion and conclusions

Brucella is one kind of intracellular parasitic bacteria, which is gram-negative bacteria. The common types of brucella that infects people are B.melitensis, B.abortus and B.suis, and each type causes diseases with different severity. The B.melitensis and B.suis would be more serious. Eating unpasteurized dairy products is a major cause of infection. The main pathogenic mechanism is that a variety of virulence factors released by the bacteria invade the host cells and evade the immune clearance of the host body, so that brucella can survive in the host cells, replicate and reproduce, and then enter the organs and tissues through macrophages to form infectious foci or migratory foci [5]. This child was infected after drinking diseased goat’s milk, and suffered from multiple system damage. The clinical symptoms were severe.

The most common symptoms of brucellosis in adults are joint pain, fever, fatigue, sweating, weight loss, myalgia, and tremor, while fever, fatigue, bacteremia, abnormal level of liver enzyme, and hepatosplenomegaly are the main clinical manifestations of brucellosis in children [6]. In this case, fever, keratitis, nervous system involvement and coronary aneurysm were the main clinical manifestations.

The incidence of neurobrucellosis is 0.5–25%, clinical presentation includes meningitis, meningoencephalitis, meningovascular involvement, parenchymatous dysfunction, peripheral neuropathy, radiculopathy, and various degrees of behavioral abnormalities [7, 8]. Neurobrucellosis is one type of inflammation caused by direct bacterial action and the effects of cytokines and endotoxins on peripheral nerves, spinal cord, meninges, brain or vascular structures [9–13]. Common signs include meningeal irritation, hyporeflexia or hyperreflexia, signs of cranial nerve involvement, positive pathological reflexes, abnormalities in sensory and motor systems to varying degrees, signs of cerebellar dysfunction, and disturbance of consciousness. The diagnosis of neurobrucellosis is based on a positive cerebrospinal fluid culture or any titration of brucella antibodies and abnormal cerebrospinal fluid measurements in the cerebrospinal fluid (cerebrospinal fluid cell count>10 × 10^6^/L, glucose decrease, protein increase) [14–16]. Although culture is the gold standard for the diagnosis of neurobrucellosis, the positive rate of culture is quite low (< 15%) [17] and takes long time. In this case, brucella was not cultured in cerebrospinal fluid, but detected by mNGS with test results available within 3 days. Therefore, it is speculated that this test can be used as an effective test for diagnosing neurobrucellosis. Neurobrucellosis has a good prognosis, with mortality reduced to 0–5.5% after appropriate antibiotic treatment. The results showed that the combined application of doxycycline, rifampicin and ceftriaxone was the best for the treatment of neurobrucellosis patients [18].

Cardiovascular involvement is the leading cause of death from brucellosis, and the most common manifestations of which are endocarditis, peripheral and cerebrovascular aneurysms or arteriovenous thrombosis [19]. The diagnosis of brucellosis with coronary aneurysms has not been reported. The patient of this case was admitted due to fever, red eyes and coronary artery aneurysm, which had been suspected to be incomplete kawasaki disease. But the child was still febrile after treatment according to Kawasaki disease. After the diagnosis of brucellosis was confirmed, the coronary aneurysm was considered to be vasculitis caused by brucellosis. Therefore, aspirin, warfarin, and dipyridamole were added to the anti-brucellosis treatment, and the symptoms of coronary artery injury gradually reduced. Therefore, we speculated that if brucellosis is combined with vasculitis in other parts, anticoagulant drugs can be added according to the degree of vascular involvement to relieve local vascular symptoms and reduce cardiovascular adverse events.

In this case, the eyes were obviously affected, indicated by keratitis and papillary edema. The ultrasound showed a slight thickening of the optic nerve in both eyes. We speculated that such phenomenon was caused by (1) neurobrucellosis caused optic neuritis, and (2) vasculitis caused by brucellosis. At present, the possible pathogenesis of neurobrucellosis induced optic neuritis are (1) Brucella-induced optic nerve and retinal nourishing vascular inflammation, and (2) Brucella infection induces an autoimmune response in the body.

According to the experience of this case, the clinical manifestations of brucellosis are diverse, which is easily confused with nervous system infection, incomplete kawasaki disease and other diseases. Therefore, when the children with fever of unknown causes have a history of suspicious exposure, relevant examination of brucellosis must be improved. Children suspected to be diagnosed with brucellosis can be tested by mNGS of brucellosis in relevant body fluids (blood and cerebrospinal fluid) as soon as possible. In particular, as a new method for pathogen detection, mNGS can produce fast results with high reliability (Additional file 1).

## Supplementary information

**Additional file 1.**

## Data Availability

The datasets used during the current study are available from the first author upon reasonable request.

## Abbreviations

PLT: Platelet
Hb: Hemoglobin
HIV: Human immunodeficiency virus
ALT: Alanine aminotransferase
AST: Aspartate aminotransferase
LCA: Left coronary arter
RCA: Right coronary arter
AO: Aortic root
LAD: Left anterior descending coronary artery

## References

[1] Rossetti CA, Arenas-Gamboa AM, Maurizio E (2017). Caprine brucellosis: a historically neglected disease with significant impact on public health. PLoS Negl Trop Dis.

[2] Bundle DR, Brucellosis MGJ (2017). Improved diagnostics and vaccine insights from synthetic glycans. Acc Chem Res.

[3] Wang H, Xu WM, Zhu KJ, Zhu SJ, Zhang HF, Wang J, Yang Y, Shao FY, Jiang NM, Tao ZY, Jin HY, Tang Y, Huo LL, Dong F, Li ZJ, Ding H, Liu ZG. Molecular investigation of infection sources and transmission chains of brucellosis in Zhejiang, China. Emerg Microbes Infect. 2020;9(1):889–99. 10.1080/22221751.2020.1754137. 10.1080/22221751.2020.1754137 PMC7241503 32284015

[4] Bosilkovski M, Stojanov A, Stevanovic M, Karadzovski Z, Krstevski K (2018). Impact of measures to control brucellosis on disease characteristics in humans: experience from an endemic region in the Balkans. Infect Dis (Lond).

[5] Hashimoto D, Miller J, Merad M (2011). Dendritic cell and macrophage heterogeneity in vivo. Immunity..

[6] Buzgan T, Karahocagil MK, Irmak H, Baran AI, Karsen H, Evirgen O, Akdeniz H (2010). Clinical manifestations and complications in 1028 cases of brucellosis:a retrospective evaluation and review of the literature. Int J Infect Dis.

[7] Zange S, Schneider K, Georgi E, Scholz HC, Borde JP (2019). A headache with surprising outcome: first case of brucellosis caused by brucella suis biovar 1 in germany. Infection.

[8] Karsen H, Tekin Koruk S, Duygu F, Yapici K, Kati M (2012). Review of 17 cases of neurobrucellosis: clinical manifestations, diagnosis and management. Arch Iran Med.

[9] Akdeniz H, Irmak Ö, Demiröz AAP (1998). Central nervous system brucellosis: presentation diagnosis and treatment. J Infection.

[10] Bucher A, Gaustad P, Pape E (1990). Chronic neurobrucellosis due to Brucella melitensis. Scand J Infect Dis.

[11] Karsen H, Akdeniz H, Karahocagil MK, Irmak H, Sünnetçioğlu M (2007). Toxic-febrile neurobrucellosis, clinical findings and outcome of treatment of four cases based on our experience. Scand J Infect Dis.

[12] Turkoglu SA, Halicioglu S, Sirmatel F, Yildiz M, Yildiz N, Yildiz S (2018). Vasculitis and neurobrucellosis: evaluation of nine cases using radiologic findings. Brain Behav.

[13] Alqwaifly M, Al-Ajlan FS, Al-Hindi H, Al Semari A (2017). Central nervous system Brucellosis granuloma and white matter disease in Immunocompromised patient. Emerging Infect Dis.

[14] Haji-Abdolbagi M, Rasooli-Nejad M, Jafari S, Hasibi M, Soudbakhsh A (2008). Clinical and laboratory findings in neurobrucellosis: review of 31 cases. Arch Iran Med.

[15] Guven T, Ugurlu K, Ergonul O, Celikbas AK, Gok SE, Comoglu S, Dokuzoguz B (2013). Neurobrucellosis: Clinical and diagnostic features. Clin Infect Dis.

[16] Sanchez-Sousa A, Torres C, Campello MG, Garcia C, Parras F, Cercenado E, Baquero F (1990). Serological diagnosis of neurobrucellosis. J ClinPathol.

[17] Ge Y, Guan HZ, Fan SY, Zhu R, Ma XJ (2017). Li TS. A clinical analysis of 20 patients with neurobrucellosis. Zhonghua Nei Ke Za Zhi.

[18] Al-Sous MW, Bohlega S, Al-Kawi MZ, Alwatban J, McLean DR (2004). Neurobrucellosis: clinical and neuroimaging correlation. AJNR Am J Neuroradiol.

[19] Herrick JA, Lederman RJ, Sullivan B, Powers JH, Palmore TN (2014). Brucella arteritis: clinical manifestations, treatment, and prognosis. Lancet Infect Dis.

