# Supplementary material for: Meningoencephalitis, coronary artery and keratitis as an onset of brucellosis: a case report
Source: BMC Infect Dis. 2020 Sep 7;20:654. doi: 10.1186/s12879-020-05358-z (PMC7487788; doi:10.1186/s12879-020-05358-z)
Supplement: Supplementary file 1 — Additional file 1. [file 12879_2020_5358_MOESM1_ESM.pdf]

CARE
case report guidelines

CARE Checklist of information to include when writing a case report

BY-NC-ND

Topic	Item	Checklist item description	Reported on Line
Title	1	The diagnosis or intervention of primary focus followed by the words "case report"	page1 (paragraph1-2)
Key Words	2	2 to 5 key words that identify diagnoses or interventions in this case report, including "case report"	page2 (paragraph3)
Abstract (no references)	3a	Introduction: What is unique about this case and what does it add to the scientific literature?	page1 (background)
	3b	Main symptoms and/or important clinical findings	page1 (case presentation)
	3c	The main diagnoses, therapeutic interventions, and outcomes	page1 (case presentation)
	3d	Conclusion—What is the main "take-away" lesson(s) from this case?	page2 (conclusion)
Introduction	4	One or two paragraphs summarizing why this case is unique (**may include references**)	page5 (paragraph2-3)
Patient Information	5a	De-identified patient specific information.	page2 (case presentation)
	5b	Primary concerns and symptoms of the patient.	page2 (case presentation)
	5c	Medical, family, and psycho-social history including relevant genetic information	N/A
	5d	Relevant past interventions with outcomes	N/A
Clinical Findings	6	Describe significant physical examination (PE) and important clinical findings.	page2 (case presentation)
Timeline	7	Historical and current information from this episode of care organized as a timeline	N/A
Diagnostic Assessment	8a	Diagnostic testing (such as PE, laboratory testing, imaging, surveys).	page3(case presentation)
	8b	Diagnostic challenges (such as access to testing, financial, or cultural)	page3(case presentation)
	8c	Diagnosis (including other diagnoses considered)	page3(case presentation)
	8d	Prognosis (such as staging in oncology) where applicable	page4(case presentation)
Therapeutic Intervention	9a	Types of therapeutic intervention (such as pharmacologic, surgical, preventive, self-care)	page4(case presentation)
	9b	Administration of therapeutic intervention (such as dosage, strength, duration)	page4(case presentation)
	9c	Changes in therapeutic intervention (with rationale)	page4(case presentation)
Follow-up and Outcomes	10a	Clinician and patient-assessed outcomes (if available)	page4(case presentation)
	10b	Important follow-up diagnostic and other test results	page4(case presentation)
	10c	Intervention adherence and tolerability (How was this assessed?)	N/A
	10d	Adverse and unanticipated events	N/A
Discussion	11a	A scientific discussion of the strengths AND limitations associated with this case report	page6(paragraph1-3)
	11b	Discussion of the relevant medical literature **with references**.	page5-6
	11c	The scientific rationale for any conclusions (including assessment of possible causes)	N/A
	11d	The primary "take-away" lessons of this case report (without references) in a one paragraph conclusion	page6(paragraph3)
Patient Perspective	12	The patient should share their perspective in one to two paragraphs on the treatment(s) they received	N/A
Informed Consent	13	Did the patient give informed consent? Please provide if requested	Yes ☑ No ☐
